# Breeding for resistance to maize streak virus: challenges, progress and future directions: a review

**DOI:** 10.3389/fpls.2025.1590870

**Published:** 2025-06-03

**Authors:** Malven Mushayi, Hussein Shimelis, John Derera, Seltene Abady Tesfamariam

**Affiliations:** ^1^ African Centre for Crop Improvement (ACCI), College of Agriculture, Engineering and Science (CAES), University of KwaZulu-Natal, Scottsville, Pietermaritzburg, South Africa; ^2^ Seed Co, Rattray Arnold Research Station, Chisipite, Harare, Zimbabwe; ^3^ One Consultative Group on International Agricultural Research (CGIAR)-Ibadan and International Institute of Tropical Agriculture (IITA), Ibadan, Oyo State, Nigeria

**Keywords:** maize streak virus, plant diseases, quantitative trait loci, resistance breeding, sub-Saharan Africa

## Abstract

Maize (*Zea mays* L.) is a commodity crop sustaining livelihoods and economies globally. However, maize productivity is challenged by many factors. Maize streak virus disease (MSV) is the most damaging in sub-Saharan Africa (SSA). It causes grain yield losses of up to 100% when susceptible varieties are grown without protection. MSV also affects the quantity and quality of crop biomass and silage production. Therefore, there is a need for effective MSV control strategies to minimize both crop yield and quality losses. Breeding and deploying MSV-resistant varieties is the most sustainable, cost-effective, and amenable control measure, especially for smallholder growers. Hence, breeding for MSV resistance in maize varieties targeted for the smallholder sector in SSA is an integral component of most breeding programs in the region. The aim of this review is to document the challenges posed by MSV, management options, breeding approaches, and progress, as well as provide recommendations and future directions. To gain insight into the host-pathogen interaction for parental selection and breeding, the first section of the paper discusses the impact, biology, host range, symptoms and epidemiology of MSV. The second section reviews breeding progress and research gaps in new variety design with MSV resistance as part of the product profiles. The paper reveals the breeding sources of genetic variation, quantitative trait loci, major- and minor-effect genes for MSV resistance and the disease control in maize. Finally, the review highlights the conventional and modern breeding methods, innovations and prospects for MSV resistance breeding. The review would guide scientists and maize breeders in developing and deploying MSV-resistant maize varieties.

## 1 Introduction

Maize is a global food security and commercial crop. In Africa, maize is cultivated on 42 million hectares with annual yield outputs of 97 million tonnes and accounting for 8% of global production (Tarekegne et al., 2024). In sub-Saharan Africa (SSA), it is the primary source of food and income for millions of households (Abebe, 2024). SSA countries have the highest per capita annual human consumption of maize globally. Over 100 million resource-poor farmers in SSA depend on maize production (Woomer et al., 2023). The consumption per capita of maize is highest in Southern Africa, exceeding 80 kg per capita per year in Lesotho, Malawi, Zambia, Zimbabwe and South Africa (Ranum et al., 2014), compared to the global average of 18.5 kg per capita per year (Erenstein et al., 2022).

The global food demand is expected to double by 2050 due to urbanisation, population pressure and lifestyle changes (Ekpa et al., 2019; Van Dijk et al., 2021). To meet global demand, a yield gain of up to 2.4% per year is needed for major food crops including maize (Mushayi et al., 2020; Chivasa et al., 2021). Maize productivity can be enhanced by breeding high-performing hybrids with desirable product profiles. However, recent declines in yield gains have been reported due to a plethora of biotic and abiotic stresses associated with climate change, threatening food security and value chains (Kim and Lee, 2023; Njeru et al., 2023).

The global average of yield of maize is 6 t/ha (Erenstein et al., 2022; Gunundu et al., 2023; Woomer et al., 2023). The southern African region is the primary producer of maize in SSA, of which South Africa is the leading producer, with a mean grain yield of 4.67 t/ha (USDA, 2024). Nevertheless, maize yields in the rest of the Southern African countries is low (<1.5 t/ha) (Mushayi et al., 2020; Erenstein et al., 2022). As a result of low maize yield there is a wide gap between production and consumption. The yield gap is the primary cause of food insecurity and malnutrition in SSA.

The maize streak virus (MSV), is listed among the major foliar diseases of the crop in SSA (Krishna et al., 2023). It causes devastating yield losses reaching 100% in susceptible cultivars especially under low and erratic rainfall (O’Halloran et al., 2024; Afram et al., 2024; Benjamin, 2024). MSV is caused by a member of Mastrevirus belonging to the family Geminiviridae (Martin and Shepherd, 2009; Claverie et al., 2023). The disease is endemic in Africa and its islands, extending to tropical and temperate zones. There is a need for effective MSV control strategies to minimise losses and boost productivity. These include the use of crop protection chemicals to control the vector transmitting MSV (Djomo et al., 2022; Mrope and Kigodi, 2024), the use of cultural practices (Aza et al., 2023), biological methods (Moya-Raygoza, 2024) and growing MSV resistant varieties (Benjamin, 2024; Sime et al., 2021; Garoma et al., 2024). Therefore, integrative MSV management spearheaded by a resistance breeding program is the overriding consideration in SSA.

The *Msv1* gene is the primary source of MSV resistance in most maize cultivars, but dependence on this single gene risks breakdown of host resistance (Garcia-Oliveira et al., 2020; Sime et al., 2021). Some moderate- and minor-effect putative quantitative trait loci also confer durable MSV resistance (Nair et al., 2015; Ladejobi et al., 2018; Sime et al., 2021). MSV resistance can be enhanced through gene stacking and integrating minor-effect QTL with major-effect *Msv1*.

The body of knowledge on the key issues relevant to pre-breeding and breeding for MSV resistance is sparse and scattered. In light of the above background, this review aims to document the challenges posed by MSV, management options, breeding approaches, and progress, as well as provide recommendations and future directions to guide resistance breeding and cultivar release and commercialisation. The first section discusses the impact, biology, host range, symptoms and epidemiology of MSV to gain insight into the host-pathogen interaction for parental selection and breeding. The second section presents breeding success stories and research gaps in new variety design and deployment with MSV resistance and desirable product profiles. The paper summarises the sources of genetic variation and hitherto reported quantitative trait loci, major- and minor-effect genes for MSV resistance breeding and disease control. Finally, the review highlights the conventional and modern breeding methods, innovations and prospects for MSV resistance breeding. The review would guide maize breeders in developing and deploying MSV-resistant and market-preferred maize varieties.

## 2 The impacts of MSV on maize production

Maize streak virus (MSV), is one of Africa’s most important viral diseases limiting maize production and economic development in SSA. MSV is cosmopolitan and transmitted by the leafhopper vector, *Cicadulina mbila* Naude (Hemiptera: Cicadellidae) (Afram et al., 2024). Epidemics of MSV is affected by environmental factors that influence leafhopper population sizes and activity. Drought conditions and irregular rains at the beginning of crop growing seasons are common triggers for MSV outbreaks (Abebe, 2024; Benjamin, 2024). Low altitudinal conditions (Iseghohi et al., 2024), high temperatures (above 24°C), low rainfall (Benjamin, 2024) and low humidity (Zulfiqar et al., 2010) influence the abundance of *Cicadulina* species with varying transmission abilities. Climate change predictions show increased precipitation in East Africa while a decrease in Southern Africa (Taye and Dyer, 2024). This leads to a favourable temporal overlap of seasons, allowing the occurrence of MSV and impacting maize productivity.

MSV infection, incidence, disease development, severity and impact depend on crop growth stage, cultivar susceptibility and growing environment. A susceptible maize genotype would fail when infected with a virulent strain of MSV, notably before it reaches the third leaf stage (Ameen et al., 2022). This leads to a 100% yield loss (O’Halloran et al., 2024; Afram et al., 2024; Iseghohi et al., 2024). Epidemic proportions of MSV have been reported in more than 20 African countries, including Togo, Benin, Burkina Faso, Ghana, Nigeria, Cameroon, Sao Tome, Uganda, Ethiopia, Sudan and Zimbabwe (Ketsela et al., 2022; Abebe, 2024). Martin and Shepherd (2009) reported a yield penalty of approximately 45% when susceptible maize genotypes were infected at the fourth leaf stage, while Djomo et al. (2021) pinpointed 5% to 14% lower grain weight under MSV infection than an uninfected control. In susceptible genotypes, a 71% yield loss was reported compared to a 10% loss by moderately tolerant and 1.5% by tolerant genotypes (Bosque-Pérez et al., 1998). MSV severity negatively correlates with grain yield-related traits such as plant height, 1000-grain weight, ear length and ear diameter (Bosque-Pérez et al., 1998).

MSV reduces the nutritional value of maize cultivars for food and feed. Iseghohi et al. (2024) reported that MSV infection can significantly decline total carotenoid content, reducing the nutritional value of pro-vitamin A maize genotypes. They also reported poor plant populations and husk cover in varieties with high MSV scores. Lukuyu et al. (2013) reported a significant reduction in crop biomass in maize in Kenya due to the early crop infection by MSV. Low biomass production is accompanied by severe leaf chlorosis and stunting, typical disease symptoms (Benjamin, 2024). Low biomass production directly affects silage production. Gross margin economic analysis showed that maize growers lost up to 0.36 tonnes/ha due to MSV early infection when cultivating susceptible genotypes (Lukuyu et al., 2013; Abebe, 2024). Therefore, the impact of MSV depends on the crop growth stage, with earlier infection leading to increased disease severity and reduced yield. Martin and Shepherd (2009) reported an annual monetary loss of 120 to 480 million USD in SSA due to lost productivity, high cost of production, and price fluctuations inflicted by MSV (Tatineni and Hein, 2023). The authors opined half of this loss could be recovered with effective MSV control strategies. The cost of MSV can be viewed in terms of the benefits it could bring with effective control strategies. The diverse and potential benefits include improved grain yield and quality, lower consumer prices and increased income for maize farmers. Farmers using MSV-resistant varieties in Kenya realised an estimated increase in crop yields by 5 to 20% per year (Martin and Shepherd, 2009). The gain is between 4 million and 15 million USD per year to subsistence farmers and a corresponding decrease in maize prices by 0.7 to 2.6%, thus saving consumers between 6 million and 17 million USD annually. Lukuyu et al. (2013) also estimated savings of 1000 USD/ha if livestock farmers adopt MSV-tolerant silage hybrids. MSV is a significant threat to maize production and productivity, adding to African subsistence farmers’ already precarious social and economic instability. Therefore, there is a need for sustainable MSV control strategies to circumvent yield and monetary loss.

## 3 The biology and host range of MSV

### 3.1 Genome description of MSV

MSV is a geminivirus with a circular, single-stranded DNA (ssDNA) molecule of about 2.7 kb (Bennett et al., 2024), encapsulated in 22 × 38 nm geminate composed of a single 32-kDa capsid protein (Shepherd et al., 2010; Tennant et al., 2018). Its sedimentation coefficients (S) are 76S and 54S for bi-segmented and single particles, respectively (Bock, 1982). The MSV genome contains two intergenic regions: the short intergenic region (SIR) and the long intergenic region (LIR). The SIR contains polyadenylation and termination signals necessary for RNA-replication and transcription (Martin, 2007). This enables the geminivirus to produce new viral particles and infect host cells. The LIR contains divergent RNA polymerase II-type promoters and other regulatory elements crucial for expressing complementary and virion sense genes (Martin, 2007). The LIR also contains sequence elements necessary for RNA replication, including an inverted repeat sequence that forms a stable hairpin loop structure (Bennett et al., 2024).

The genome is also composed of four open reading frames, which are bidirectional transcribed and translated to produce the replication (Rep) proteins (Rep and Rep A), coat protein and movement protein (Lazarowitz, 1988; Lazarowitz and Beachy, 1999). The Rep and Rep A proteins interact with each other and various host factors, directing the host cell to form replication complexes required for virus DNA replication (Shakir et al., 2023). The coat protein is a multifunctional protein essential for various stages of the virus life cycle, including DNA encapsulation, transmission, and systemic infection (Brown, 2008). The movement protein mediates the cell-to-cell movement of the virus (Hehnle et al., 2004).

### 3.2 MSV strains

MSV has 11 major strains designated as MSV-A to MSV-K (Monjane et al., 2011; Karavina, 2014), of which MSV-A causes the most severe disease in maize (Fouad et al., 2024; Oyeniran et al., 2024). In addition to maize, the 10 strains infect other cereal crops such as barley (*Hordeum vulgare)*, wheat (*Triticum aestivum*), oats (Avena sativa), rye (*Secale cereale*), sugarcane (*Saccharum officinarum*), pearl millet (*Pennisetum glaucum*) and finger millet (*Eleusine coracana*) (Tennant et al., 2018). Shepherd et al. (2010) and Abebe (2024) reported that the host range of MSV includes the following 14 genera of plants: *Dactylocterium, Euchlanaena, Eleusine, Paspalum, Sporobolus, Eragrostis, Imperata, Brachiara, Diplachne, Rottboelia, Setaria, Tragus, Leptochloa* and *Coix*.

### 3.3 MSV transmission and host range

MSV is transmitted by leafhoppers, small insects of the Order Homoptera, primarily the vector *C. mbila* (Djomo et al., 2021). The leafhoppers acquire the virus particles from phloem sap of infected host plants during feeding (Turan et al., 2020; Wang and Blanc, 2021). The virus then undergoes circulative, non-propagative transmission. It crosses the midgut, entering the hemolymph, and reaching the salivary glands, where it’s secreted into the saliva and transmitted to the new host plant (Wang and Blanc, 2021; Dixcy, 2022). Once a leafhopper takes up the virus, it takes about two days for the virus to reach its salivary glands (Martin and Shepherd, 2009). In this transmission mode, viral acquisition, latency, and inoculation periods range from several hours to days, while the virus can be retained in the vector for several days to weeks (Hogenhout et al., 2008; Vilanova et al., 2022).

Leafhoppers feed on over 80 species of monocotyledonous plants that belong to the Poaceae family (Malar et al., 2023). *Cicadulina* spp. also feed on various plant hosts, including veld paspalum (*Paspalum orbiculare*), common signal grass (*Brachiara villosa*), napier grass (*Pennisetum purpureum*), rapoko grass (*Eleusine indica*) and creeping crabgrass (*Digitaria horizontalis*).

### 3.4 Symptoms of maize streak virus

**Figure 1:**
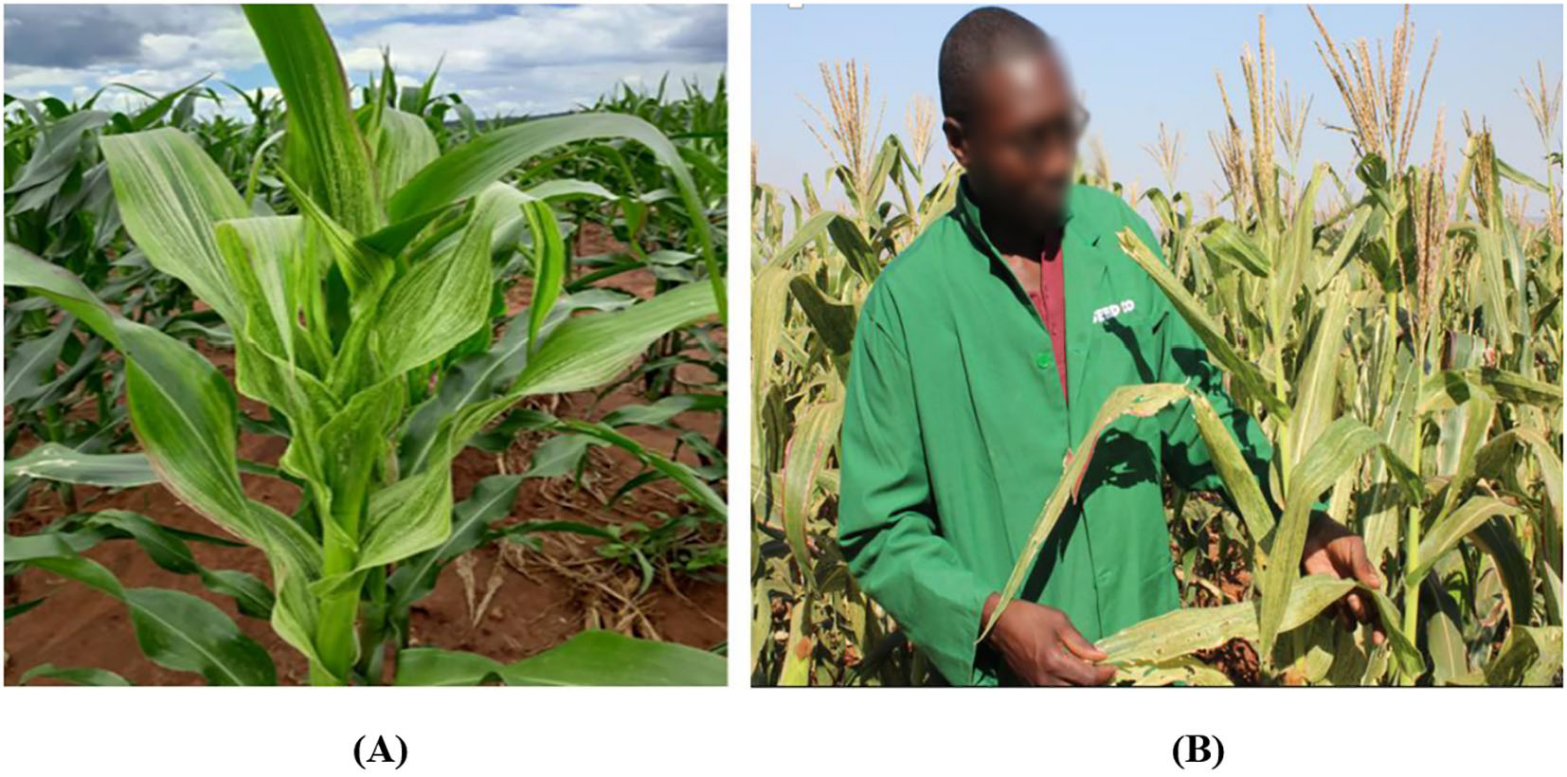
Maize plant showing chlorotic symptoms caused by Maize Streak Virus infection at the Muzarabani Research Station **(A)** and at the Rattray Arnold Research Station site **(B)** in Zimbabwe (Photo supplied by Malven Mushayi, 2024).

Understanding the MSV infection, symptoms and disease development is crucial for developing effective management strategies, including resistance breeding. The distribution of MSV can be sporadic. However, it causes a devastating yield and biomass loss and crop failure under epidemic proportions. **Figure 1** shows typical MSV chlorotic symptoms in maize field in Zimbabwe. The virus causes various symptoms in susceptible genotypes, including broken or continuous leaf chlorotic streaks along the primary, secondary and tertiary veins and stunted plant growth (Benjamin, 2024). Leaf chlorosis inhibits photosynthesis, cell division, plant growth and development and ultimately, grain yield and quality losses (Lata-Tenesaca et al., 2024; Xu et al., 2024).

The streaks initially appear as small, pale, circular spots on the youngest leaves. Sime et al. (2021) and Iseghohi et al. (2024) indicated that newly formed leaves show signs of MSV infection, while old leaves below the infection point may appear healthy. As the disease progresses, the spots cause pale yellow lines up to several millimetres long down the leaf blades, parallel to the veins. In highly susceptible genotypes, chlorotic streaks merge to form uniform chlorosis. Leaf chlorosis is triggered by the failure of chloroplast to develop in the tissues around the vascular bundles (Monjane et al., 2020).

Stunted plant growth is a quantifiable MSV infection symptom in susceptible genotypes (Jiang and Zhou, 2023; O’Halloran et al., 2024). Cell division is negatively impacted by MSV infection, leading to leaf twisting, corrugation or curling (Monjane et al., 2020). Young maize plants infected within three weeks of emergence experience severe stunting, producing abnormal cobs and smaller grains and impacting grain yield and quality (Shepherd et al., 2010; Rodier et al., 1995). Infection that occurs eight weeks after planting may not cause yield damage and crop loss (Page et al., 1999).

### 3.5 Epidemiology of MSV

MSV has erratic epidemiology across years or seasons. It may have minimal impact in some seasons/years and destroy crops in others. The disease appears yearly or less frequently, depending on the virulence and composition of the virus strains, the population size and activity of the vector, crop cycle, occurrence of alternate hosts such as wild grass and environmental factors (Oyeniran et al., 2024).

Fouad et al. (2024), reported that MSV-A is the most widely distributed strain, leading to economic losses. The authors indicated that this strain has five variants (i.e., MSV-A1, MSV-A2, MSV-A3, MSV-A4 and MSV-A6). MSV-A1 is the most widely distributed and virulent strain in southern, western, central and eastern Africa, menacing maize production. MSV-A probably evolved through the breakdown of MSV tolerance genes in the common maize varieties (Shepherd et al., 2010). The dynamic evolution of MSV-A’s virulence in maize necessitates breeding a new generation of maize varieties in the region. The MSV-A is assumed to have diverged from MSV-B, its nearest non-maize adapted relative (van der Walt et al., 2008; Harkins et al., 2009). Reportedly, the MSV-A-like virus underwent genetic recombination with another virus strain, MSV-F, producing the most damaging MSV-A that occurs today (Shepherd et al., 2010; Fouad et al., 2024). Mixed infection by MSV-A and MSV-B strains are common. Genetic recombination between the diverse virus strains is a unique mechanism to break down genetic resistance in dispatched maize genotypes (Monjane et al., 2020; Fouad et al., 2024).

The biology, mobility and dispersal behaviour of the leaf hopper species influence the epidemiology of MSV. *Cicadulina mbila* is the most efficient transmitter of maize streak virus compared with other *Cicadulina* species in Africa (CABI, 2022). It is the most widely distributed vector species throughout Africa compared with other *Cicadulina* species (Storey, 2008; Fouad et al., 2024). Female *C. mbila* occur widely and are effective MSV transmitters (Shepherd et al., 2010). The proliferation and mobility of leaf hoppers affect the efficiency of MSV transmission. In warm and wet seasons, *C. mbila* develops a longer body morph, which flies less than 10 m, causing low disease incidence (Shepherd et al., 2010). The short-bodied *C. mbila* types are fast flyers and conquer many fields, especially during crop maturity and drought conditions. The vectors migrate extensively into irrigated crops, causing severe MSV incidence and crop damage.

Successive crop cycles per season boost the *C. mbila* populations and MSV infection. The disease occurs in early planted maize, damaging seedling plants of ultra-short season hybrids and hosting the vector as a bridge to the next crop. A second crop following the ultra-early hybrids, such as wheat during the winter season, also hosts leafhoppers, creating a temporary bridge for leafhopper survival and perpetuating the MSV infection cycle (Shepherd et al., 2010). Infection is exacerbated when susceptible ultra-short-season hybrids are planted at high population density, which promotes leafhopper proliferation and virus spread. In addition, shorter growing seasons also limit corrective action and recovery, as timely insecticidal control may not be feasible. Some farming practices are also associated with increased MSV incidence. For example, multiple maize plantings in a single growing season can increase MSV incidence, with late-planted crops generally experiencing higher infection rates than early planted crops (Martin and Shepherd, 2009). Monoculture and the expansion of maize production also areas aggravate MSV incidence in Africa (Chivasa et al., 2020).

Although maize is the preferred host for *Cicadulina*, several grass species have also been implicated in leafhopper abundance and MSV incidence. Leafhoppers breed on over 138 grass species, with 70% of these being potential MSV hosts (Varsani et al., 2009). Shepherd et al. (2010) found that the maize-adapted MSV-A strain and its close relative, the grass-adapted MSV-B strain, are particularly virulent on plants of the genus *Digitaria.* After mating, fertilized leafhoppers prefer grass species for oviposition.

MSV also infects other grass species, such as wheat (*Triticum aestivum*), oats (*Avena sativa*), rye (*Secale cereal*), barley (*Hordeum vulgare*), and sugarcane (*Saccharum officinarum*). Of these, sugarcane streak disease, caused by the MSV-A strain, is the most prevalent and devastating in sugarcane crops in southern Africa (Varsani et al., 2009).

The epidemiology of MSV is influenced by the environmental conditions that favour the leafhopper and its fecundity. Studies in Kenya revealed a significant correlation between climate factors and the damage caused by MSV, highlighting the influence of climate on the spread and prevalence of the disease (Magenya, 2008). The authors indicated that the late onset of rainfall creates an environment conducive to the growth and development of leafhopper nymphs during the winter months, resulting in a surge in vector population. Also, leafhoppers migrate to green areas during low humidity and high temperatures. It thrives in temperatures between 20-30°C. Effective management of MSV requires an integrated approach, including resistance breeding, accurate disease forecasting, and an understanding of the epidemiology of MSV, seasonal and yearly patterns, virulence, and vector dynamics. This will be crucial for developing effective integrated pest management strategies.

## 4 Management strategies for MSV

The major MSV control strategies include cultural practices, chemical control, biological control, host resistance and integrated disease management practices. **Table 1** presents various control measures for MSV and briefly elaborated on below.

**Table 1:** Reported management methods of MSV and its vector in maize production.

Method	Country	Description of the method	References
Cultural practices	Benin	Intercropping of maize with cassava or groundnuts significantly reduced vector population and resulted in lower MSV infection rates.	Aza et al. (2023)
	Cameroon	Intercropping of maize with cassava and or soybean reduced leafhopper activity and MSV infection rates.	Djomo et al. (2022)
	Ghana	Intercropping of maize with beans or millet significantly reduced vector activity and disease incidence by 14.9% and 17.4%, respectively.	Page et al. (1999)
		The application of potassium fertilizer reduced MSV severity by stimulating the biosynthesis of flavonoids and phenolic acids.	Magenya (2008); Blankson et al. (2018)
Chemical control	Cameroon	Imidacloprid applied at 500g in 2L water per 100kg maize seed provided 14 days of residual protection.	Chipole et al. (2016); Djomo et al. (2022)
	Italy	Lambda-cyhalothrin was applied as a contact pesticide and effectively controlled leafhoppers for several weeks.	Tirello et al. (2021)
		Acetamiprid systemic pesticide effectively controlled early and older leafhopper nymphs.	
Use of botanicals	Cameroon	Neem seed oil, a biological extract of *Azadirachta indica* L, applied at a 4ml/litre of water significantly reduced the vector population and maintained MSV infection below 5%.	Djomo et al. (2022)
		Leaf extracts of Mexican tea or wormseed (*Dysphania ambrosioides*), applied at 6.7 mL per litre of water, reduced vector activity and infection rates.	
Biological control	Mexico	The leafhopper egg is parasitized by earwigs (*Doru linear*), anagras (*Anagrus virlai T*) and parasitic wasps (*Paracentrobia subflava* Girault and *Pseudoligosita* sp).	Virla et al. (2015); Moya-Raygoza (2024)
		Solitary wasp (*Gonatopus bartletti*) and assassin bugs (*Zelus obscuridorsis*) are the parasites of leafhopper nymphs and adults.	Moya-Raygoza (2024)
Integrated disease management	South Africa	A combination of cultural practices (e.g. crop rotation, removal of weeds and early planting), MSV-resistant varieties, and chemical strategies effectively controlled MSV.	Collins and Duffy (2022)
	Ghana	Integrating biological control, cultural control (rouging of infected plants), MSV-resistant varieties, and chemical control were effective in controlling MSV.	Seidu et al. (2022)
		The integration of four control methods, including biological control, rouging of infected plants, host resistance and chemical control, was useful in controlling MSV.	Ackora-Prah et al. (2023)

### 4.1 Cultural practices

Cultural practices are aimed at reducing vector movement, multiplication and MSV transmission to susceptible maize varieties and non-host crops (Tatineni and Hein, 2023). The common cultural practices used by growers include crop rotation, intercropping with non-host crops, removal of crop residues and weeds, rouging diseased plants, adjusting planting dates, early planting (Martin and Shepherd, 2009). Additionally, maintaining good soil health through balanced soil nutrition is also beneficial (Blankson et al., 2018).

Intercropping maize with major crops minimises MSV vectors. Aza et al. (2023) reported that intercropping maize with cassava or groundnuts significantly reduced the vector population, and resulted in lower MSV infection rates in Benin. In Cameroon, maize-cassava and maize-soybean intercrops markedly reduced vector activity (Djomo et al., 2022). Further, intercropping maize with common beans and millet in Ghana reduced vector and disease incidence by 14.9% and 17.4%, respectively (Page et al., 1999). Magenya (2008) and Blankson et al. (2018) reported that adequate soil potassium can reduce MSV severity in maize, probably by stimulating the biosynthesis of compounds, such as flavonoids and phenolic acids, which induce antiviral defence responses.

Cultural practices are essential MSV avoidance strategies notably used by smallholder farmers but may not be sustainable when used alone. The nature of MSV epidemiology and leafhopper populations make it challenging to detect the peak MSV transmission periods. Moreover, smallholder farmers without irrigation facilities are compelled to plant during the onset of rains, rendering early planting strategies unfeasible (Martin and Shepherd, 2009). Unpredictable weather patterns and altered rainfall distributions make uncertain planting decisions. Additionally, small land holdings (0.5-1.5 hectares per household) and the high demand for maize products hinder effective crop rotation under the smallholder condition in Africa (Karavina, 2014). Roguing diseased plants has little effect because insect vectors can quickly re-infect healthy plants. Cultural control strategies are more effective when combined with MSV-tolerant or resistant cultivars (Shepherd et al., 2010).

### 4.2 Chemical control

Insecticides suppress MSV by targeting the leafhopper vector. Systemic insecticides, including neonicotinoids such as imidacloprid, acetamiprid, thiacloprid, and nitenpyram, are used as a seed dressing or applied at the early growth stage (Mrope and Kigodi, 2024). This protects the initial crop stand and plant development. Tirello et al. (2021) reported that acetamiprid significantly reduced early and older leafhopper nymphs. Imidacloprid has been effective against leafhoppers when maize seed was treated at 500g per 2 litres of water per 100kg grain of maize (Chipole et al., 2016). Imidacloprid disrupts insect reproduction and egg development, with prolonged residual activity of 14 days after application (Djomo et al., 2022). Contact insecticides, such as Lambda-cyhalothrin, a pyrethroid that interacts with the nervous system of the pest (Farag et al., 2021), are essential for complementing neonicotinoids, which have short-duration protection. Tirello et al. (2021) reported that a single application of lambda-cyhalothrin significantly reduced leafhopper population densities for several weeks in Italy.

The use of crop protection chemicals has several challenges. Firstly, pesticides are unaffordable for most resource-poor farmers. Furthermore, they pose significant health risks to farmers and the environment due to their high toxicity and repeated application requirements (Karavina et al., 2014; Alam et al., 2016; Afram et al., 2024). Inadequate application techniques can lead to poor pest control, harm to non-target species and pose an increased risk of pesticide poisoning (Babendreier et al., 2019; Wirasti et al., 2021). Moreover, insecticides offer partial control protection when disease pressure is severe (Chivasa et al., 2020). Exclusive reliance on pesticides can also lead to the development of pesticide-resistant leafhopper populations, which can be challenging for adequate crop protection (Adegorite et al., 2024; Khan et al., 2024). Chemical control strategies are more effective in integrated pest and disease management systems.

### 4.3 Biological control methods

Various biological agents including earwigs (*Doru lineare*, Insecta: Dermaptera: Forficulidae) (Virla et al., 2015), anagras (*Anagrus virlai*, Triapitsyn; Hymenoptera: Mymaridae) and parasitic wasps including *Paracentrobia subflava* Girault (Hymenoptera: Trichogrammatidae) (Moya-Raygoza, 2024) can suppress leafhoppers. However, the sustainable use of biological control agents is challenged by limited availability, specificity to leafhopper biotypes (Magenya, 2008), and potential harm to non-target species and ecosystems (Hoddle, 2024).

Alternatively, botanical extracts such as neem seed oil extracted from the neem species (*Azadirachta indica* L) and leaf extracts of Mexican tea or wormseed (*Dysphania ambrosioides*, formerly *Chenopodium ambrosioides* L), have shown efficacy in controlling leafhoppers. These extracts have advantages including reduced risk of insecticide resistance, low environmental persistence, and cost-effectiveness (Munyoki et al., 2024; Khan et al., 2024). However, they may also have variable efficacy, low toxicity, and persistence, and can be affected by environmental factors (Guo et al., 2024).

### 4.4 Host plant resistance

Host resistance offers a sustainable and cost-effective solution to control MSV under small-holder or commercial farmers. The option provides a durable and effective means of disease management. Adopting MSV-resistant cultivars allows farmers to boost crop resilience and grain yields with minimal costs and labour requirements (Benjamin, 2024). This approach is less dependent on frequent insecticide applications, minimizing environmental pollution and chemical residues (Straub et al., 2020). Moreover, host resistance is a long-term solution that can be maintained for multiple seasons, making it a viable option for small-scale farmers (Abebe, 2024).

MSV resistance breeding involves identifying and incorporating major and minor resistance genes into high-yielding maize varieties (Garcia-Oliveira et al., 2020). Conventional breeding methodologies, including recurrent selection (Kolawole et al., 2019), backcrossing, pedigree selection (Abalo et al., 2009) and hybrid breeding (Ige et al., 2023) have been deployed to develop MSV-resistant varieties. MSV resistance breeding delivered genetically diverse, improved populations with enhanced grain yields and tolerance to MSV (Menkir et al., 2024; Mukaro et al., 2024).

Elite inbred lines were deployed for MSV resistance breeding. The notable MSV-resistant elite maize inbred lines developed included CML202 (Welz et al., 1998), Tzi3, Tzi4, Tzi15, Tzi17 (Efron et al., 1989), TZEI-7, TZEI-22 (Ige et al., 2017), IB32 (Emeraghi et al., 2021). These lines were used in population improvement and hybrid breeding programs. Successful hybrids such as C92 x CML202, EM11-133 x CML202, CML197 x CML202 and C92 x EM11-133 with high-yielding and MSV-resistance were developed using CML202 (Gichuru and Njoroge, 2011).

Other MSV-resistant hybrid varieties developed through conventional breeding included SC403, SC627, Pan 4M-19, MH 26, MH 27, MH 28 (Haraman, 2016), PAN53, PAN6549, MRI514, PRIS601, MRI514 and MRI744 (Nhantumbo et al., 2021),16XH71, 16XH24, 16XH31 (Maphumulo et al., 2021) and 053WH54 (Karavina et al., 2014; Mukaro et al., 2024). Also, open-pollinated varieties with MSV resistance were bred, such as TZECOMP3DT, MVDC2SYNF2, DTSRWC2, EV97DTSTRW (Emeraghi et al., 2021), Matuba, Chinaca, Tsangano and ZM523 (Nhantumbo et al., 2021), AK-9528-DMRSR, Acr. 91 Suwan-1-Sr C1, IK.91 TZL Comp 3-Y C1, Ikenne 88 TZSR-Y-1, and TZB-SR SGY TZB-SR (Bello, 2017; Ige et al., 2023). The developed genetic resources were shared with national research programs in various countries, including Cameroon, Ghana, Malawi, Tanzania, Uganda and Zambia (Krishna et al., 2023) for further selection, yield stability tests, and eventually release. **Table 2** presents various MSV-resistant/tolerant maize germplasm and their gene banks.

**Table 2:** Description of reported maize genetic resources and gene banks as sources of variation for MSV resistance breeding.

Genetic resources	Name/designation	Gene bank	Trait description	Reference
Landraces	Tuxpeño Planta Baja	IITA/Nigeria	MSV tolerant	Bedoya et al. (2017); Nelimor et al. (2019)
Open pollinated varieties	ACR06		Showed a lower MSV infection rate of 12%	Djomo et al. (2021)
	MADJSYN VAR2		Showed a lower MSV infection rate of 13.3%	
	TZSR-W/Y		MSV tolerant	Emeraghi et al. (2021)
	TZESR-Y			
	AK-9528-DMRSR, Acr. 91 Suwan-1-Sr C1, IK.91 TZL Comp 3-Y C1, Ikenne 88 TZSR-Y-1, and TZB-SR SGY TZB-SR.		Good general combiner for MSV resistance	Ige et al. (2023); Bello (2017)
	Tigli	CSIR/South Africa	Resistant to MSV, rust, leaf blight, *Curvularia* leaf spot, and stalk lodging	CORAF (2019)
	KASSAI	IRAD/Cameroon	Showed a low infection rate of 5%	Djomo et al. (2021)
	ATP		Showed a low infection rate of 14%	
	ZM 309	CIMMYT/Malawi	MSV/Tolerant	Haraman (2016)
	TZECOMP3DT	CIMMYT/Benin IITA/Benin, INRAB/Benin	MSV and drought-tolerant	Emeraghi et al. (2021)
	MVDC2SYNF2			
	DTSRWC2			
	EV97DTSTRW			
	Longe 1 and Longe 6H	CIMMYT/Tanzania	MSV tolerant	
	Matuba, Chinaca and Tsangano	IIAM/Mozambique		Nhantumbo et al. (2021)
	ZM523	CIMMYT/Mozambique		
Elite lines	TZi3	IITA/Nigeria	Donors for MSV resistance breeding	Efron et al. (1989)
	TZi4			
	TZi5			
	Tzi17			
	TZE1-7			Ige et al. (2017)
	TZE1-22			
	IB32			Emeraghi et al. (2021)
	TZE117		Recommended as a tester for MSV resistance	Allen et al. (2020)
	CML202	CIMMYT/Tanzania	With major QTL (*Msv1*) mapped on chromosome 1, the line CML202 has been used by several researchers as a donor and tester for MSV resistance.	Welz et al. (1998)
	CML390		MSV tolerant lines useful for hybrid breeding and population improvement.	Nyaligwa et al. (2017)
	CML505			
	SML129			
	MAS[MSR/312]-119-5-1-1-3-B			
	WPop x 1368 STR S7			
	CML442		Recommended as testers for MSV resistance breeding.	Allen et al. (2020)
	CML444			
Wild relatives	*Zea diploperennis*		Donors for MSV, maize chlorotic dwarf virus, maize chlorotic and mottle virus resistance breeding.	Wani et al. (2022)
Mutants	Obatampa derivative mutant lines	CSIR/South Africa	Lines used to develop high-yielding MSV-resistant hybrids.	Afram et al. (2024)
Synthetics	HGA	IITA/Nigeria	Donor parents to improve provitamin A content and MSV tolerance.	Iseghohi et al. (2024)
	HGB			
	STR Syn.-W		Tolerant to MSV and *Strigga*	FAO (2008)
	STR Syn.-Y			
	STR Syn.-Y/W			
	Syn 4		Good general combiner for MSV and leaf blight resistance	
	TZL COMPOSITE		Donor of MSV resistant genes	
Composites	TZE COMPOSITE 3		Tolerant to MSV	
	TZL COMPOSITE 5			
	TZE COMP 4	IITA/Togo		
	VHCw	UCT/South Africa	Donors of MSV resistance genes.	(van Rensburg, 2005)
	VHCy			
	WH 502			Emeraghi et al. (2021)
Modern hybrids	PAN67	PANNAR/South Africa	MSV tolerant	Lukuyu et al. (2013)
	G31			Nyaligwa et al. (2018)
	G23			
	G18			
	G22			
	SC403			Haraman (2016)
	SC627			
	PAN53	PANNAR	MSV tolerant	Emeraghi et al. (2021)
	MH 27			
	MH 28			
	Pan 4M-19			
	MRI514	Syngenta/Mozambique	MSV tolerant	Nhantumbo et al. (2021)
	MRI744			
	PRIS601	K2/Mozambique	MSV tolerant	
	Namuli	IIAM/Mozambique	MSV tolerant	
	053WH54	CBI/Zimbabwe	MSV resistant	Karavina et al. (2014); Mukaro et al. (2024)
	16XH71	UKZN/South Africa	MSV resistant	Maphumulo et al. (2021)
	16XH24			
	16XH31			
Obsolete cultivars	SC407, SC411, SC621, SC635, SC715, SC717, and SC721	Seed Co. Ltd/Zimbabwe and Zambia	Old MSV resistant hybrids	Seed Co. Ltd (2007)

MSV, Maize Streak Virus; IITA/Nigeria, International Institute of Tropical Agriculture/Nigeria; KALRO, Kenya Agricultural and Livestock Research Organization; CIMMYT/Kenya, International Maize and Wheat Improvement Center/Kenya, CIMMYT/Malawi, International Maize and Wheat Improvement Center/Malawi; CIMMYT/Benin,International Maize and Wheat Improvement Center/Benin; IITA/Benin, International Institute of Tropical Agriculture/Benin; INRAB/Benin, Institut National des Recherches Agricoles du Bénin; CIMMYT/Tanzania, International Maize and Wheat Improvement Center/Tanzania; K2/Mozambique, Klein Karoo/Mozambique; IIAM/Mozambique, Agricultural Research Institute of Mozambique, CBI/Zimbabwe, Crop Breeding Institute/Zimbabwe; UKZN/South Africa, University of KwaZulu Natal/South Africa; CSIR/South Africa, Council for Scientific & Industrial Research/South Africa; IRAD/Cameroon,Institute of Agricultural Research and Development/Cameroon; CIRAD/Kenya, Agricultural Research Centre for International Development/Kenya; IITA/Togo, International Institute of Tropical Agriculture/Togo; UCT/South Africa, University of Cape town/South Africa.

Modern breeding technologies, including genomic-assisted selection, genetic engineering, and gene editing, have been integrated with conventional breeding methods. These tools aided in the breeding and deploying of high-yielding and MSV resistance inbred lines such as T2 and T3 and maize single cross hybrids such as T2Hi-II x WM3 (Shepherd et al., 2007a) and MSV-resistant three-way maize hybrids, including CML444/CML312//L3, CML444/CML395//L4 and CML444/CML312//L9 (Abalo et al., 2009).

### 4.5 Integrated MSV management

The use of one or a few control strategies may not guarantee complete protection against MSV (Collins and Duffy, 2022). Integrated disease management (IDM), which combines at least two complementary disease control methods, is the most effective approach (Mrope and Kigodi, 2024; Pokharel et al., 2024). For example, Collins and Duffy (2022) found that combining cultural practices (e.g., removing or burning weeds), MSV-resistant varieties, and crop protection chemicals significantly reduced MSV infections more effectively than using each method alone. In addition, Seidu et al. (2022) used cultural practices (e.g. rouging of infected plants), MSV-resistant varieties, and chemical control to reduce MSV infections. Ackora-Prah et al. (2023) reported that the optimal MSV management strategy is integrating biological control, rouging of infected plants, host resistance, and chemical control.

Implementing IDM strategies has its pros and cons. For instance, smallholder maize growers have limited knowledge of MSV infection, disease development and plant-virus interactions to monitor the disease (Farwah et al., 2020). Furthermore, IDM integrates several time-consuming methods that demand regular monitoring, coordination, and control (Huded et al., 2023). In addition, the method requires adequate finance, which smallholder farmers may be unable to afford. Including MSV-resistant varieties in most of the IDM strategies demonstrates the importance of host resistance in MSV management.

## 5 MSV resistance breeding of maize

### 5.1 Conventional

Plant breeding aims to develop genetically enhanced crop cultivars with economic benefits for growers and the market place (Dossa et al., 2023). Conventional breeding is the most widely used method for developing disease-resistant varieties, especially in developing countries (Garoma et al., 2024). The method mainly involves creating new genetic variations, selecting desirable genotypes in the progeny and stability analysis and commercialisation. Genetic variation can be harnessed using existing germplasm (e.g. landraces, crop wild relatives, open-pollinated varieties, obsolete varieties and expired plant variety protected lines) or generated artificially through controlled crossing, polyploidization and induced mutations (Dossa et al., 2023). Large-scale germplasm screening for disease resistance through artificial inoculation or natural infection in hotspot areas enables the identification and breeding of resilient varieties (Garoma et al., 2024).

The most commonly used conventional breeding methods for MSV resistance include hybrid breeding, pedigree breeding, backcrossing, and recurrent selection. These approaches offer the advantage of (in) direct selection for environmental adaptation (Abalo et al., 2009). In addition, these methods can be carried out using local resources, expertise and tools (Lamichhane and Thapa, 2022). Significant progress has been made in developing high-yielding MSV-resistant maize hybrids using conventional breeding methods. However, conventional breeding approaches rely on selection based on the phenotype of the plants, which is influenced by genotype, environment and genotype x environment interaction effects. Classical approaches are also time-consuming and may not keep pace with the rapid emergence of new and virulent disease races and strains and insect biotypes. This can lead to the breakdown of major resistance genes (Abalo et al., 2009). Therefore, modern molecular tools (e.g. marker-assisted breeding, genetic engineering, and gene editing) should be integrated to complement conventional breeding methods (Chandra et al., 2024).

#### 5.1.1 Components of MSV resistance

Host plant resistance to MSV comprises three components: antibiosis, antixenosis, and tolerance, which collectively work to control leafhoppers and MSV in maize (Purnomo, 2021).

##### 5.1.1.1 Antixenosis

Antixenosis, or non-preference, is a natural defence mechanism that prevents leafhoppers from feeding, oviposition, or sheltering on maize plants (Nalam et al., 2019). This mechanism reduces initial colonization and leafhopper population size, thereby decreasing MSV spread. As a primary line of defence, antixenosis is crucial in maize, specifically deterring the leafhopper vector from transmitting MSV (Singh et al., 2021).

Resistant maize varieties may possess increased levels of phosphorus, potash (Saleh et al., 2024), lignin, and cellulose (Santiago et al., 2013). These compounds repel leafhoppers, reduce feeding and oviposition, and interfere with pest mouthpart function. Certain maize inbred lines, such as E739, CML206, P606, P590, P612, and CML202, exhibit antixenosis, of these E739 and CML206 show strong resistance (van Rensburg, 2001). Similarly, resistant varieties such as 8321-21 display reduced leafhopper probing, increased non-feeding activities and shorter phloem feeding duration (Mesfin and Bosque-Perez, 1998). This indicates strong antixenotic resistance compared to the susceptible variety, FR114 x FR303. Other MSV-tolerant maize genotypes, including 100 MSR and HASR, detered *Cicadulina mbila* from settling, probing, and ovipositing compared to the susceptible H512 genotype (Magenya, 2008).

##### 5.1.1.2 Antibiosis

Antibiosis is a plant defence mechanism that disrupts the leafhopper’s life cycle by affecting growth, development, reproduction, and survival (Purnomo, 2021). This mechanism is mediated by the plant’s production of polyphenols, a diverse group of compounds comprising flavonoids, phenolic acids, stilbenes, and lignans. These compounds affect leafhopper survival by binding with enzymes and proteins, inhibiting digestion, growth and development (Singh et al., 2021). Specifically, maize varieties such as Lamuru and Arumba, with high phenolic content, exhibit toxicity to leafhoppers compared to susceptible varieties in Indonesia (Saleh et al., 2024).

In addition, antibiosis also effectively reduces viral replication within plants. Maize varieties exhibiting elevated levels of enzymes, particularly phenylalanine ammonia-lyases, accumulate salicylic acid, a crucial compound disrupting the viral pathogenicity cycle (Purnomo, 2021). Salicylic acid inhibits viral replication, cell-to-cell movement, and long-distance movement within the plant (Cueto-Ginzo et al., 2016). Researchers use genetic engineering methods to further enhance resistance to develop transgenic maize with improved antibiosis against MSV. The MSV replication-associated protein gene (*rep gene*) has been the primary target for gene silencing due to its crucial role in enhancing viral replication. Successful approaches include using spliceable-intron hairpin RNA (*hpRNA*) to silence the *rep gene*, resulting in MSV-resistant transgenic varieties (Owor et al., 2011). Transgenic maize inbred lines designated as T2, T3, and single cross hybrid, T2Hi-II x WM3, have demonstrated enhanced antibiosis under MSV challenge through *rep gene* silencing (Shepherd et al., 2007a).

##### 5.1.1.3 Tolerance

Tolerance to MSV enables maize plants to withstand leafhopper attacks and MSV infection without significant yield loss (Benjamin, 2024). Maize genotypes display varying degrees of tolerance to MSV, conditioned by their inherent ability to utilise soil nutrients efficiently (Faisal, 2015) and genetic factors that regulate recovery capacity (Emeraghi et al., 2021). For instance, Obatanpa, an improved maize variety released in Ghana, outperformed the local variety Domabin, in yield and MSV resistance due to its nutrient use efficiency, particularly potassium absorption (Blankson et al., 2018). Enhanced potassium levels boost disease tolerance by reducing nutrient competition and increasing phenol concentrations (Wang et al., 2013). MSV-tolerant maize genotypes can recover from infection and compensate for lost growth (Purnomo, 2021). This recovery-type resistance is marked by reduced symptom severity, low virus titers, and maintained productivity (Sime et al., 2021). In a study by Ladejobi et al. (2018), maize lines initially showed susceptibility to MSV infection in the first few weeks and recovered with emerging healthy upper leaves enabling growth and photosynthesis.

The genetic constitution of the host underlying recovery resistance are mediated by gene interactions. The *Msv1* QTL reportedly conferred varying levels of MSV resistance (Garcia-Oliveira et al., 2020).

#### 5.1.2 Genetic variation for MSV resistance breeding

The availability of genetic diversity is paramount for successful maize breeding, which focuses on MSV resistance and desirable product profiles. The primary sources of genetic variation for maize breeding and product development include landraces (Bedoya et al., 2017), open-pollinated varieties (Nhantumbo et al., 2021), elite breeding lines (Allen et al., 2020), crop wild relatives (Wani et al., 2022), mutants, synthetic varieties (Nelimor et al., 2019), composites/multilines (van Rensburg, 2005), modern hybrids (Nhantumbo et al., 2021), obsolete cultivars (Dossa et al., 2023) and expired plant variety protected lines (Dao et al., 2020; McCluskey and Tracy, 2024). **Table 2** summarize reported maize landraces, open-pollinated varieties, elite breeding lines, crop wild relatives, mutants, synthetic varieties, composites, modern hybrids, obsolete cultivars and expired plant variety protected lines and their gene banks as sources of variation for MSV resistance breeding.

Landraces or farmers’ varieties are dynamic populations maintained under the prevailing growing conditions. Landrace populations of maize are characterized by higher levels of genetic diversity and heterogeneity and local adaptation, making them ideal sources of genetic variation for breeding (Caldu-Primo et al., 2017; Dossa et al., 2023). Landraces can be tapped to develop crops with desirable traits such as high grain yield and enhanced resilience to biotic and abiotic stresses (Allen et al., 2020; Masoni et al., 2020; Santos et al., 2024). Maize landraces conserved by the International Plant Genetic Resources Institute (IPGRI) constitute a valuable gene pool for yield improvement (Dossa et al., 2023). A collection of 196 maize landraces were characterized by Nelimor et al. (2019), representing a diverse gene pool from Burkina-Faso, Ghana, and Togo for breeding for higher grain yield, disease tolerance, ear prolificacy, ear length and kernel row number per ear. Meseka et al. (2013) reported that maize landrace collections from the Northern Guinea Savanna and Sudan Savanna in West and Central Africa have shown some drought-adaptive traits. Maize landraces are potential gene donors for MSV resistance breeding programs. For instance, the Tropical Zea Yellow (TZY) population, derived from the landrace Tuxpeño Planta Baja (Bedoya et al., 2017; Nelimor et al., 2019), has been a foundational genetic resource for developing MSV-resistant maize inbred lines. Researchers from IITA have successfully generated MSV-resistant lines from landraces, including Tzi3, Tzi4, Tzi15 and Tzi17 (Efron et al., 1989) and IB32 (Emeraghi et al., 2021). Furthermore, Tigli, an open-pollinated and MSV-resistant maize cultivar, was developed from a landrace variety in Ghana (CORAF, 2019).

Open-pollinated varieties of maize are predominantly cross-fertilisers and maintained through population breeding. OPVs show extensive genetic diversity and possess novel alleles for breeding (Masuka et al., 2017). OPVs with resistance to MSV exhibiting higher genetic gains of 192.9 kg ha−1 yr−1 (early maturity types) and 108.7 kg ha−1 yr−1 (late maturity groups) were reported in SSA by CIMMYT (Masuka et al., 2017). Superior OPVs, notably Acr. 91 Suwan-1-SR C1 possessing a desirable combination of MSV resistance, ear prolificacy, high grain carotenoids and protein contents, and high grain yield were used to develop MSV resistant top cross hybrids, including TZEQI 82 × ACR. 91 SUWAN-1-SR C1 in Nigeria (Bello, 2017; Ige et al., 2023). In addition, the OPV Obatanpa was used to develop MSV mutant lines in Ghana (Afram et al., 2024).

Elite inbred lines are developed through continuous selection and selfing, offering several genes in homozygous states. The genetic uniformity of lines enables accurate identification of MSV resistance genes and QTL for effective genetic recombination (Sarkar et al., 2023). Inbred lines could have complementary genetic backgrounds maintained through controlled selfing, making them suitable for heterotic breeding. High-yielding, locally adapted and market-preferred MSV-resistant maize hybrid varieties are attained through hybrid breeding involving elite inbred lines (Dossa et al., 2023). Efforts by private seed companies, public breeding programs, and international research organizations like CIMMYT and IITA have focused on developing MSV-resistant/tolerant inbred lines for use in hybrid breeding, synthetic cultivar development and population improvement (Asea et al., 2009).

Maize wild progenitors, including *Zea diploperennis, Tripsacum dactyloides, Z. mexicana*, and *Z. parviglumis* (teosinte), possess unique genetic diversity lost in the cultivated maize due to artificial selection (Wani et al., 2022). Introgressing novel alleles from wild relatives has proven successful in conferring disease resistance in cultivated maize (Dossa et al., 2023). For instance, grey leaf spot resistance was introduced in a maize population derived from the cross of *Z. parviglumis* with a maize line B73 (Wani et al., 2022). Similarly, *Z. diploperennis* from Jalisco and Mexico has been identified as a valuable source of resistance to northern and southern corn blight and multiple viral diseases, including MSV, maize chlorotic mottle virus, and maize chlorotic dwarf virus (Warburton et al., 2017; Wani et al., 2022). Yet more untapped genetic diversity is available in wild-related species for gene transfer, including MSV resistance in maize.

Induced mutagenesis has been effectively used in crop breeding to create new genetic variability by introducing stable and heritable mutations in the plant genome (Wanga et al., 2020; Dossa et al., 2023). This creates new valuable traits that can be incorporated into well-adapted cultivars. Unlike natural genetic variation, which occurs at a low frequency (10−5 to 10−8 per locus), induced mutagenesis can increase genetic variation by 1000 to a million times, making it a crucial strategy for genetic enhancement in crop breeding programs (FAO/IAEA Mutant Varieties Database; Jiang and Ramachandran, 2010; Wanga et al., 2020). Mutation breeding has improved MSV resistance, grain yield, and agronomic traits in maize. For instance, Afram et al. (2024) conducted a study where four maize genotypes (Obatampa, Dapango, Pann 54, and Honampa) were gamma irradiated at 254.3 to 300 Gy rates. The study found that M4 mutants derived from the Obatampa variety showed enhanced tolerance to MSV and produced a high grain yield of 6.8 t/ha. This corroborates the findings of Matova et al. (2021), who demonstrated that optimal gamma irradiation can improve plant height, ear height, and grain yield in maize mutants. Mutation breeding provides a cost-effective and rapid approach to enhancing genetic diversity and the development of MSV-resistant maize varieties.

Synthetic varieties, developed by intermating multiple inbreds with high general combining ability, offer a new source of genes for improving MSV resistance in maize. These populations provide significant benefits to farmers and breeders. Farmers can save seeds for subsequent crop cycles, ensuring cost-effective and sustainable commercial production (Dossa et al., 2023; Dhilllon and Jat, 2024). Synthetic varieties are valuable genetic source population for selecting new breeding lines (Emeraghi et al., 2021) and developing novel hybrid varieties, which can help reduce the breeding cycle. For instance, Iseghohi et al. (2024) studied the tolerance of provitamin-A-enriched maize to MSV under field conditions in Nigeria. The authors used three selection cycles (C0, C1, and C2) derived from maize synthetics (HGA and HGB) and their crosses. The results showed that three selections (PVASYNHGAC2, PVASYNHGBC0, and PVASYNHGBC2) and five varietal-cross hybrids exhibited high tolerance to MSV. The authors recommended using seeds from these selection cycles as donor parents to improve provitamin A and MSV tolerance in maize breeding programs. The IITA in West and Central Africa has identified Striga-resistant synthetic varieties (STR Syn.-W, STR Syn.-Y, and STR Syn.-Y/W) that exhibited promising resistance to MSV (Kim et al., 2006; FAO, 2008). These synthetic varieties were developed by intercrossing Striga- and MSV-resistant maize inbred lines, combining desirable traits from both sources. Using the STR Syn synthetics, IITA breeders developed Striga-resistant maize inbred lines, including TZISTR1011. Another synthetic variety, Syn 4, developed by IITA and available in Nigeria, combines good MSV resistance with resistance to leaf blight (FAO, 2008).

Composite varieties or multilines are developed by mixing seeds of promising lines and maintained through open pollination (Dossa et al., 2023). Their broad genetic base offers opportunities for adaptation to various environmental conditions. In West Africa, several MSV-resistant composite varieties were registered by the IITA, including TZE COMPOSITE 3, TZE COMPOSITE 5, TZL COMP 4 and TZL COMPOSITE (FAO, 2008). IITA has developed genetically diverse Striga-resistant maize inbred lines through trait introgression from some of the synthetic cultivars, as validated by Dossa et al. (2023). Notable examples include maize inbred line TZSTRI101, derived from the MSV-resistant synthetic variety TZL COMPOSITE, and TZISTR1259, TZISTR1263, TZISTR1275, TZSTRI112, TZSTRI114, and TZSTRI115, developed from the MSV-resistant synthetic variety TZE COMP5. Furthermore, TZL COMP4 was utilized to develop high-yielding and MSV-tolerant inbred lines through recurrent selection methods, providing a valuable source of genes for breeding programs (Kolawole et al., 2019). The composites designated as Vaalharts VHCw and VHCy served as donors to develop MSV-resistant inbred lines, including J2705tv, allowing improved hybrids such as SA4 and SA5 in South Africa (van Rensburg, 2005).

Obsolete maize cultivars, include outdated breeding lines, OPVs, composites and hybrids that have been replaced by newer improved versions. Despite being discontinued due to their susceptibility to emerging climate change-induced stresses or shifts in market preferences and demand, these genetic resource remain valuable, harbouring diverse alleles for key traits in maize. In Zimbabwe, obsolete OPVs released and marketed by the CBI in 1960 include Salisbury White, Hickory King and Southern Cross (Mazibuko et al., 2024). Additionally, old hybrids released by the CBI in 1971 include R200, R201, R215, and SR52, which were developed and released in 1960 (Mazibuko et al., 2024). Obsolete varieties can be used to introgress valuable genes into modern maize breeding programs. For instance, Hickory King possesses desirable traits such as superior taste, large kernel size, high kernel density, kernel hardness, and weevil-resistance (Machida et al., 2014). In addition, SR52 was the first single-cross maize hybrid to be commercialized worldwide and has formed the basis of maize breeding in Zimbabwe and the Eastern and Southern African regions (Derera and Musimwa, 2015). Gene banks conserves obsolete genetic resources. For instance, the Seed Co Ltd gene bank maintains obsolete MSV-resistant hybrids, including SC407, SC411, SC621, SC635, SC715, SC717 and SC721 (Seed Co, 2007). These old hybrids were previously marketed in Zimbabwe and Zambia but were replaced by newer, improved varieties such as SC419, SC555, SC657 and SC727 (Seed Co, 2024). The obsolete maize varieties are valuable genetic resources for breeding maize with enhanced MSV resistance.

#### 5.1.3 Screening for MSV resistance

Effective screening for MSV resistance and tolerance is crucial for selecting desirable parents and hybrid breeding. Screening for MSV resistance can be undertaken under hotspot fields or environmentally controlled testing conditions (Iseghohi et al., 2024). The two methods can be used separately or in combination to select desirable genotypes.

##### 5.1.3.1 Screening in natural hotspots

Field screening involves using natural MSV hotspots where maize is predominantly cultivated. Field sites represent the natural crop growing conditions and are characterized by favourable climatic conditions for the proliferation of the leafhopper and high MSV incidence. Reportedly, the following sites were found to be conducive for field screening: Wenchi in Ghana (Ige et al., 2017; Allen et al., 2020), Mokwa in Nigeria, (Iseghohi et al., 2024) and Ngaramtoni Research Farm in Tanzania (Nyaligwa et al., 2017), indicating their potential as cross-border germplasm evaluation. Natural screening for MSV resistance is economical and efficient in assessing many genotypes over sites and seasons (Nyaligwa et al., 2017). The main limitation of this method is infection variability and disease escape, which causes an overestimation of resistance. Field screening involving designed spreader rows was found to be successful for infection, disease development and evaluation (Abalo et al., 2009). Spreader rows consist of MSV-susceptible maize genotypes established in a border or alternate pathways with candidate genotypes to enhance inter-plot infection.

##### 5.1.3.2 Artificial screening

Artificial inoculation of test genotypes under greenhouse or controlled environment conditions limits disease escape and enables genotype discrimination based on severity (Mukaro et al., 2024). Artificial screening involves optimised viral inoculation using mass-reared leafhopper vectors (Mutengwa et al., 2012; Djomo et al., 2021). During artificial screening, vectors are released in the leaf whorl of maize plants for disease transmission. The leafhoppers are reared using non-viruliferous plant populations, commonly using pearl millet (*Pennisetum typhoides*) seedlings in insect-proof cages. Seedling plants are inoculated 10 days after sowing using two or more viruliferous leafhoppers to initiate infection and disease development (Mukaro et al., 2024).

Artificial infestation methods combined with spreader rows enable uniform virus spread across greenhouses or fields, established plants, and rows. Artificial screening have some challenges, including the high cost of establishing leafhopper-rearing and testing facilities, labour requirements, uncontrolled vector movement to non-target crops, and scalability of the method for breeding programs (Abalo et al., 2009).

##### 5.1.3.3 Rating of maize genotypes for MSV resistance

**Figure 2:**
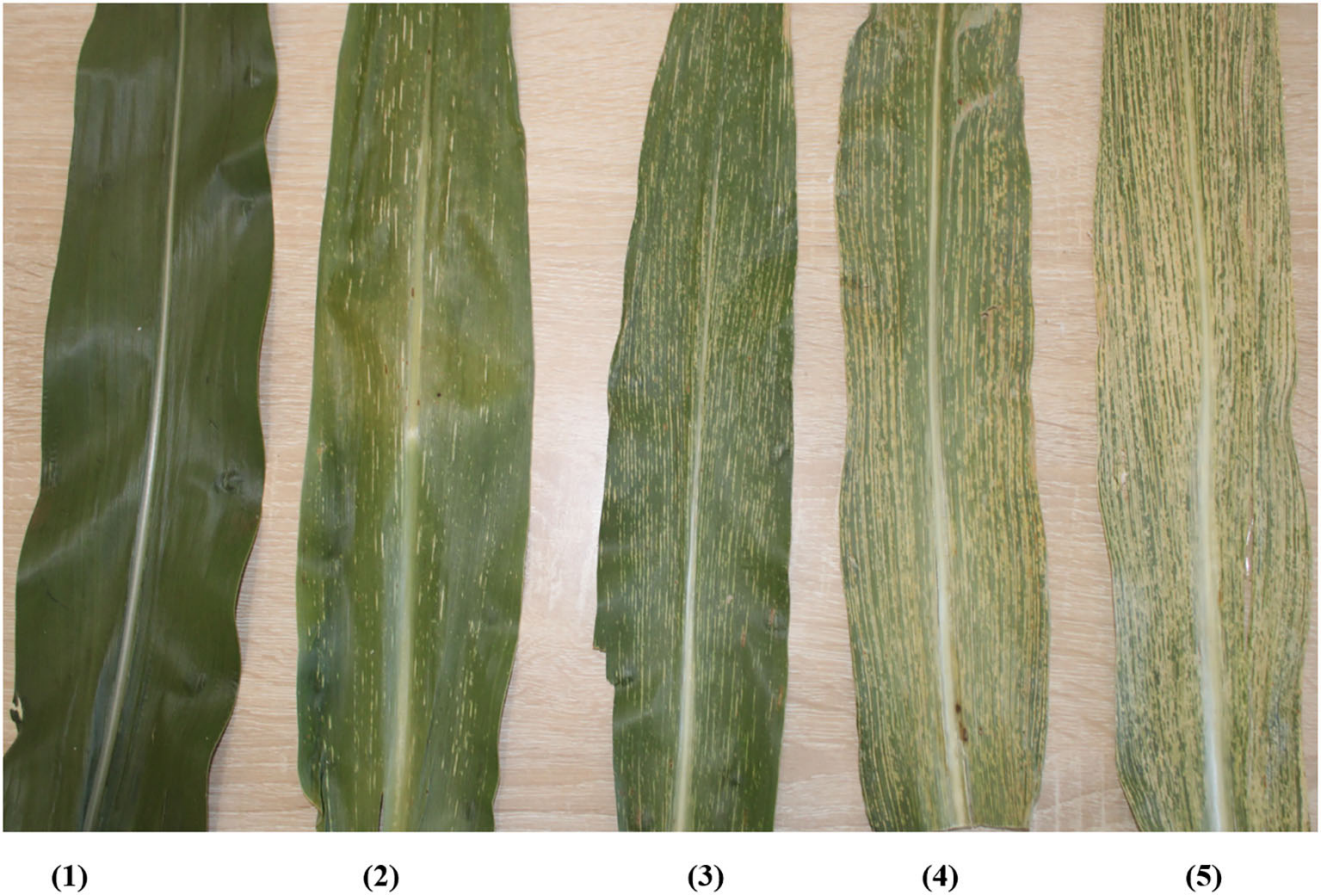
Scheme showing a rating of MSV infection using a scale of 1 to 5. Note: 1 = immune, 2 = highly resistant, 3 = resistant, 4 = moderately resistant and 5 = susceptible (Photo supplied by Malven Mushayi, 2024).

MSV disease severity is assessed and rated by visual evaluation on a semi-quantitative scale. A standardized scoring system is used to classify resistance reactions and genotypes. A disease score with a scale of 1 to 5 (Kyetere et al., 1999; Mbong et al., 2021) or 1 to 9 (Eyal et al., 1987; Chivasa et al., 2020) are used to assess genotypes for MSV severity. Based on a scale of 1 to 5, a genotype with a score of 1 shows immunity, while scores of 2, 3, 4 and 5 correspond to highly resistant, resistant, moderately resistant, and susceptible categories, respectively (**Figure 2**) (Kyetere et al., 1999; Afram et al., 2024). Alternatively, the 1 to 9 scale categorizes resistance as follows: a score of 1 is resistant, 2 to 3=moderately resistant, 4 to 6 =susceptible, and 7 to 9=highly susceptible (Eyal et al., 1987; Chivasa et al., 2020).

### 5.2 Genomic-assisted breeding in MSV resistance breeding

#### 5.2.1 Molecular markers and marker-assisted breeding

Molecular markers have enhanced MSV resistance breeding (He et al., 2024). Various molecular markers, including Simple Sequence Repeats (SSRs), Single Nucleotide Polymorphisms (SNPs), and Insertions/Deletions (InDels), have been developed for maize (Garcia-Oliveira et al., 2020). Due to the high heritability of MSV resistance, primarily attributed to major gene effects, the technology offers several advantages, including enhanced accuracy, improved efficiency, cost-effectiveness, minimized phenotyping, and enhanced genetic gains (He et al., 2024). For instance, Abalo et al. (2009) found marker-assisted selection (MAS) to be 26% cheaper and more efficient, resulting in lower MSV incidence than conventional selection (64.8% vs 79.3%). MAS led to the development of MSV-tolerant lines L3, L4, and L9, which were deployed to create high-yielding and MSV-resistant three-way maize hybrids, such as CML444/CML312//L3, CML444/CML395//L4 and CML444/CML312//L9 in Uganda (Abalo et al., 2009).

Gene stacking has been recommended to boost the frequencies of minor-effect alleles in segregating populations by integrating agronomically desirable multiple donor parents bearing minor-effect MSV resistance genes with *Msv1* gene (Joshi et al., 2024). Emeraghi et al. (2021) reviewed the breeding process, which commenced with crosses between a recurrent parent (RP) and MSV donor parent, followed by backcrossing to RP and selecting BC1F1 lines, yielding desirable genetic recombinations. Progenies with appreciable genetic recombination are then selfed, and MSV resistance genes are pyramided among selected lines, combining minor-effect QTL with the major-effect QTL (*Msv1*). Integrating marker-assisted backcrossing (MAB) in gene pyramiding schemes reportedly doubled or tripled targeted allele frequency in the new progenies (Emeraghi et al., 2021). Marker-assisted breeding facilitates a rapid advancement of progenies with desired gene combinations, resulting in new stacked lines possessing enhanced MSV resistance and desirable agronomic characteristics. Despite these benefits, linkage drag makes MAS less efficient (Vieira et al., 2025). There is a need to intergate MAS with traditional breeding methods to enhance selection response and genetic gains. Also, MAS depends solely on selecting genetic markers, independent of environmental effects, and may not be associated with the expression of MSV resistance under field conditions. Integrating MAS with conventional selection involving field evaluations enhances selection response by indirectly selecting for environmental adaptation. This combined approach ensures that developed lines are MSV-resistant and adapted to diverse environments.

#### 5.2.2 Genomic selection

Genomic selection is a marker-assisted selection technique that uses historical phenotypic data and genome-wide molecular markers to predict the performance of genetically related breeding populations (Vieira et al., 2025). GS has successfully identified maize inbred lines with resistance to major diseases such as MSV, northern corn leaf blight, and grey leaf spot (Gunundu et al., 2023).

However, the technology has not yet been fully embraced by most private and public maize breeding programmes in Africa. The integration of doubled haploid (DH) populations makes GS a crucial tool in modern breeding programs. The DH technology eliminates the need for multiple generations of inbreeding for hybrid breeding (Rice and Lipka, 2021; Matova et al., 2023). GS with DH technologies are key for speed breeding and market competitiveness. Genomic-assisted breeding ensures higher genetic gains and minimises variability attributable from genotype-by-environment (GEI) interactions (Peixoto et al., 2024).

#### 5.2.3 Quantitative trait loci analysis

Quantitative trait loci analysis has emerged as a powerful tool for marker-assisted selection to improve MSV resistance in maize breeding programs. QTL analysis has identified two major loci, *msv1* and *Msv1*, contributing to MSV resistance in maize breeding. *Msv1*, a dominant locus located on chromosome 1, confers complete resistance to MSV and accounts for 40-76% phenotypic variation in resistant lines (Welz et al., 1998; Kyetere et al., 1999; Nair et al., 2015). Fine mapping of the *Msv1* locus has delimited the region to 0.87 cM, containing the candidate gene GRMZM2G046848, a U-box domain-containing tyrosine kinase family protein. *Msv1* is detected in IITA inbred line Tzi4 and CIMMYT population CML206 × CML312 (Nair et al., 2015). *Msv1* is allelic to a recessive QTL, *msv1*, identified in the same genomic region in CIMMYT inbred line CML202 (Welz et al., 1998). The recessive *msv1* QTL confers partial resistance to MSV and regulates plant defence responses (Garcia-Oliveira et al., 2020).

The identification of both *Msv1* and *msv1* has enabled the development of new lines among maize breeding pipelines, facilitating the selection of MSV-resistant genotypes (Nair et al., 2015). Nevertheless, reliance on the single major-effect QTL, *Msv1*, is insufficient to acquire durable resistance to MSV. The mastrevirus’s high mutation rates and recombination frequency threaten the durability of MSV resistance (Emeraghi et al., 2021). This is attributable to the unpredictable nature of virus evolution and the complex, quantitative inheritance of the trait. Future strategies for achieving durability of MSV resistance in SSA maize farming systems should consider gene stacking and the integration of minor-effect QTLs that interact with the major-effect *Msv1* through epistasis. Evidence suggests that these genetic factors act together to boost resistance to MSV (Efron et al., 1989; Ladejobi et al., 2018).

The *Msv1* QTL was mapped using a large F2 population of CML206 × CML312 (Nair et al., 2015). The authors delimited the *Msv1* QTL to a 7.62 Mb interval, flanked by two SNPs (PZE-101090728 and PZE-0186365075) with a genetic distance of 0.87 cM based on two-point linkage analysis. Three KASP assays were developed from these SNPs: PZE-0186065237, PZE-101093951, and PZE-0186365075. These assays co-segregate with PZE-101090728, one of the flanking markers delimited in the *Msv1* interval, and exhibit significant association with response to maize streak virus based on haplotype trend regression (Nair et al., 2015). The KASP assays were validated and are currently used to classify phenotypes accurately based on their response to MSV (Garcia-Oliveira et al., 2020).

Sime et al. (2021) validated the diagnostic ability of the three SNPs co-segregated with PZE-101090728 in 151 early-generation IITA maize inbred lines of diverse genetic backgrounds, along with nine MSV-resistant elite lines and cultivar Pool-16, a susceptible check (Sime et al., 2021). The authors categorized maize lines into resistant, moderately resistant, susceptible, and highly susceptible by artificial inoculation of MSV using viruliferous leafhoppers under screen house conditions. Also, three SNPs associated with MSV resistance were detected in 131 candidate lines evaluated by Sime et al. (2021). The authors classified the lines into resistant (54), moderately resistant (76), and susceptible (1). The 18 early-generation lines without these SNPs were categorized as moderately resistant (10), susceptible (5), and highly susceptible (3). The study confirms the strong association between the SNP markers and MSV resistance in maize, highlighting their usefulness for forward breeding and the need for additional markers for MSV resistance screening.

Welz et al. (1998) identified a major QTL, *Msv1*, on chromosome 1 associated with putative QTL on chromosomes 2, 3 and 4, all conferring MSV resistance. Ladejobi et al. (2018) identified four QTL in 250 maize lines that significantly boosted MSV resistance, with two QTL on chromosome 3 accounting for 47 to 51% of the resistance and two others on chromosomes 7 and 9 contributing an additional 28 to 32%. Furthermore, 18 QTL were identified with moderate to minor effects on MSV resistance in maize. The genes were located on chromosomes 1, 2, 3, 4, 5, and 7 (Garcia-Oliveira et al., 2020). These QTL account for 3.1 to 21.4% of the genetic variation in MSV recovery resistance and were identified using 948 Diversity Array Technology (DarT) markers in a cross of ((KU1414 x 9450) x 9450)-15-2-1-BBB-1-B*11 and (GTMAS: Gk x KU1414SR x GT-MAS: Gk)-8-1-2-4-B*12 in an F2:3 population.

SNP loci conferring recovery resistance to MSV were identified in elite Tropical Zea Yellow (TZ-Y) maize germplasm (Ladejobi et al., 2018). The IB32 line, derived from TZY germplasm, exhibited a stable quantitative resistance to MSV, governed by additive genetic effects of 2-3 genes (Emeraghi et al., 2021). This line has been utilized in developing MSV-resistant germplasm due to its stability in time and space. The molecular basis of host-related genetic factors is yet unknown in full, but the quantitative nature of MSV resistance suggests the involvement of multiple loci with a variable magnitude of resistance. **Table 3** presents quantitative trait loci and genes responsible for MSV resistance and the gene banks of the founder genotypes.

**Table 3:** Gene banks and genotypes with quantitative trait loci and genes conditioning MSV resistance.

Source of resistance	Type of QTL/gene present	Gene bank	Reference
CML202	Major effect QTL *msv*1 gene and 7 modifying genes located on chromosomes 2, 3, 4, 5, 6, 8 and 10	CIMMYT	Welz et al. (1998)
CML204	Major effect QTL *Msv1* and 2-3 minor genes		
CML206	Major effect QTL *Msv1 and* 2 minor effects QTL		Nair et al. (2015)
D211	Major effect QTL *Msv1*, and 7 modifying genes located on chromosomes 2, 3, 4, 5, 6, 8 and 10	Réunion island	Pernet et al. (1999)
CIRAD390	Major effect QTL *Msv1* and 7 modifying genes found on chromosomes 2, 3, 4, 5, 6, 8 and 10		
IB32	Major genes, 2-3 genes	IITA/Nigeria	Efron et al. (1989)
TZIL07A01322	Recovery QTLs on Chromosome 3, 7, 9		Ladejobi et al. (2018)
TZIL07A01005	Recovery QTLs on Chromosome 3, 7, 9		
Tzi4	Major effect QTL *Msv*1 gene		Nair et al. (2015)
Arkells hickory	Single, incompletely dominant gene	South Africa	Efron et al. (1989)
Peruvian yellow			

QTL, Quantitative trait loci; CIMMYT, International Maize and Wheat Improvement Center; IITA/Nigeria, International Institute of Tropical Agriculture/Nigeria.

#### 5.2.4 Genetic engineering

A single dominant MSV resistance gene can be easily introgressed into productive susceptible varieties. This can be achieved through traditional crosses or a dedicated genetic engineering platform. The platforminvolves gene cloning and transfer using a soil bacterium (*Agrobacterium tumefaciens*) or particle bombardment method into commercially preferred maize genotypes adapted to different growing conditions (Shruti et al., 2024). Genetic engineering enables the integration of multiple genes and the development of durable MSV-resistant maize varieties through direct gene transfer and novel gene construction (Reddy et al., 2009; Hafeez et al., 2023).

Genetic engineering using Pathogen-Derived Resistance (PDR has been reported to be promising in producing MSV-resistant maize cultivars. Key viral genes were modified to disrupt MSV replication in maize cells (Martin and Shepherd, 2009). Shepherd et al. (2007b) modified the MSV replication-associated protein gene (rep) and introduced it into *Digitaria sanguinalis*, a model grass species, via particle bombardment. This resulted in transgenic plants that showed complete resistance to MSV. Shepherd et al. (2007a) also reported one defective gene halting viral replication in maize and conferring high levels of MSV resistance when transferred to maize and related grass species. They used dominant negative mutants of the MSV rep gene and developed resistance in maize. They developed transgenics, including inbred lines designated as T2 and T3 and a hybrid designated as T2Hi-II x WM3. The transgenics displayed delayed disease symptoms and development, decreased disease severity and higher plant survival rates than conventionally developed maize varieties. This was the first report on a transgenic MSV-resistant maize variety developed worldwide (Shepherd et al., 2007a).

There is still a gap in the breeding and deployment of transgenic maize resistant to MSV in Africa. Genetic engineering can deliver MSV-resistant cultivars except for the high costs, labour, and time spent in the breeding pipeline and product development. There is also, inadequate national policies and legislation, as well as public concerns regarding the safety of GM products for human, animal, and environmental health, are key obstacles in deploying MSV tolerance via genetic engineering (Nang’ayo et al., 2014). However, some African countries, including Kenya, Nigeria and South Africa, have approved the cultivation of GM maize, demonstrating a growing acceptance of this technology (Sadikiel Mmbando, 2024). Notably, in South Africa, GM maize seeds with insect and herbicide resistance genes are successfully marketed highlighting the potential economic benefits of GM crops (Shepherd et al., 2007a).

#### 5.2.5 Genome editing

Genome editing technologies enables precise and efficient modification of plant genomes without gene transfer. Gene editing offers significant advantages over conventional breeding methods, including enhanced precision, efficiency, and flexibility, making it an attractive approach for MSV resistance breeding. Various researchers explored the application of gene editing techniques to develop MSV resistance in maize, reviewing approaches such as dominant negative mutants (Shepherd et al., 2007a; Yoon et al., 2020; Xia et al., 2021) and inducible transgene expression (Shepherd et al., 2014).

Dominant negative mutants of the viral rep gene effectively combat MSV by disrupting the virus’s replication mechanism through genetic modification (Yoon et al., 2020). This technique introduces mutated forms of the rep gene that interfere with forming functional viral protein complexes, inhibiting viral replication and infection (Xia et al., 2021). Furthermore, an inducible transgenic approach has been utilized to express a resistance gene encoding a protein triggered in response to MSV infection, conferring additional viral resistance (Shepherd et al., 2014).

Cutting-edge gene editing techniques such as Clustered Regularly Interspaced Short Palindromic Repeats -associated protein (CRISPR/Cas) system has been successfully used to edit the genomes of various plants, including tobacco, Arabidopsis, and rice. For instance, edited tobacco varieties resistant to yellow dwarf virus and geminiviruses related to MSV were reported (Talakayala et al., 2022). Furthermore, engineered guide RNAs (gRNAs) have been successfully delivered into plants to target and confer resistance to pea early browning and tobacco rattle viruses through gene editing (Talakayala et al., 2022; (Ali et al., 2018). Additionally, gRNAs homologous to wheat dwarf virus were delivered into rice cells via CRISPR-Cas, achieving 19.4% knock-in frequency in transgenic plants (Talakayala et al., 2022; Ali et al., 2018). These tools offer valuable applications in MSV resistance breeding, allowing for targeted editing and removal of susceptibility factors from maize chromosomes. Genome editing in Africa faces obstacles, including regulatory uncertainty, limited research infrastructure, and inadequate biosafety and biosecurity frameworks (Abkallo et al., 2024).

## 6 Conclusion and outlook

MSV devastates maize production, causing up to 100% yield loss, reduced nutritional value, and significant economic losses. Breeding MSV-resistant genotypes is the most sustainable and economical option when combined with other methods. Therefore, it must be prioritized. Understanding the various components of MSV resistance enables breeders to identify and utilize valuable MSV genetic resources from diverse sources. Conventional breeding methods have played a significant role in MSV resistance breeding. However, the rapid evolution of mastrevirus limits the development of durable MSV resistance in maize varieties when relying solely on conventional breeding methods. Modern breeding techniques offer powerful approaches to develop superior MSV-resistant cultivars quickly. There is a need to integrate advanced breeding tools in the maize breeding programs to facilitate rapid identification and selection of promising lines possessing MSV resistance, high-yielding potential, good agronomic performance and farmer-preferred traits.
